# Repurposing of substances with lactone moiety for the treatment of γ-Hydroxybutyric acid and γ-Butyrolactone intoxication through modulating paraoxonase and PPARγ

**DOI:** 10.3389/fphar.2022.909460

**Published:** 2022-07-22

**Authors:** Sepand Tehrani Fateh, Amir Salehi-Najafabadi

**Affiliations:** ^1^ School of Medicine, Shahid Beheshti University of Medical Sciences, Tehran, Iran; ^2^ Department of Microbiology, School of Biology, University College of Science, University of Tehran, Tehran, Iran

**Keywords:** gamma hydroxybutyric acid, gamma butyrolactone, addiction, paraoxonase, peroxisome proliferator-activated receptors gamma, drug repurposing

## Abstract

GHB and GBL are highly accessible recreational drugs of abuse with a high risk of adverse effects and mortality while no specific antidotes exist. These components can also be found in the clinical setting, beverages, and cosmetic products, leading to unwanted exposures and further intoxications. As the structural analogue of GABA, GHB is suggested as the primary mediator of GHB/GBL effects. We further suggest that GBL might be as critical as GHB in this process, acting through PPARγ as its receptor. Moreover, PPARγ and PON (i.e., the GHB-GBL converting enzyme) can be targeted for GHB/GBL addiction and intoxication, leading to modulation of the GHB-GBL balance and blockage of their effects. We suggest that repurposing substances with lactone moiety such as bacterial lactones, sesquiterpene lactones, and statins might lead to potential therapeutic options as they occupy the active sites of PPARγ and PON and interfere with the GHB-GBL balance. In conclusion, this hypothesis improves the GHB/GBL mechanism of action, suggests potential therapeutic options, and highlights the necessity of classifying GBL as a controlled substance.

## Introduction

γ-Hydroxybutyric acid (GHB) and γ-Butyrolactone (GBL) are recreational drugs of abuse with easy accessibility and low cost but high risk of adverse effects and mortality (P Busardo and W Jones, 2015). These drugs are categorized as central nervous system depressants (Wong et al., 2004). Direct abuse of GHB and GBL might not be as prevalent as other drugs; however, they possess a steep dose-response curve and a narrow safety margin between a recreational dose and a fatal dose; hence, overdoses can quickly become life-threatening while no specific antidotes are available (P Busardo and W Jones, 2015). GHB is classified as a schedule I controlled substance in the USA and schedule III or IV in the EU nations (P Busardo and W Jones, 2015). In contrast, the classification of GBL as a controlled substance is controversial due to its extensive unavoidable applications (P Busardo and W Jones, 2015). In addition to the recreational usage of GHB and GBL, individuals can be exposed to these components though other routes, such as the application of GHB in the clinical setting. Moreover, GHB and GBL are produced in the process of wine production, leading to concerns about its unwanted intoxication (Addolorato et al., 1998; Elliott and Burgess, 2005). GBL has also found a place in cosmetic and industrial applications such as the production of nail polishes (Rambourg-Schepens et al., 1997), batteries (Belov and Shieh, 2012; Papović et al., 2017; Shi et al., 2017; Papović et al., 2018), and solar cells (Venkatesan et al., 2015), increasing the chance of exposure and further intoxication. In addition, GBL possesses therapeutic properties for certain clinical settings, such as alcohol addiction (Fadda et al., 1983) or seizures (Depaulis et al., 1988), while its level in the brain is related to anesthesia and induction of sleep (Bessman and Skolnik, 1964); which might act as potential causes of intoxication. GBL has also been identified in the liquid from Virginia Tobacco flavored pods (Holt et al., 2021).

GHB is considered the structural analogue of GABA (P Busardo and W Jones, 2015) and acts on GABA receptors directly, while a family of GHB receptors exists, which regulate GABA-ergic activities (Corkery et al., 2015). Following the oral administration of GHB, the absorption and distribution of the drug occur quickly, leading to behavioral manifestations in about 15 min (Galloway et al., 1997). Vomiting, ataxia, no gag reflex, acute delirium, confusion, agitation, hypothermia, clonic muscle movements, respiratory depression, coma, sudden altered states of consciousness, and death are the typical sign and symptoms of GHB overdose, and many patients will require intubation (P Busardo and W Jones, 2015; Krasowski, 2019). The clinical features of GHB and GBL toxicity are discussed in (Liechti and Kupferschmidt, 2004; Liechti et al., 2006; Wood et al., 2011). As no effective antidotes exist, supportive care is remained as the only option to save the patients (Schep et al., 2012). However, further investigations on the enzymes involved in the activation/deactivation of GHB and GBL, such as PON and their regulatory pathways, might lead to a treatment option for overdosed patients.

GHB is also an endogenous metabolite of γ-Aminobutyric acid (GABA) found in mammalians (Brennan and Van Hout, 2014). GHB and GBL can readily transform into each other through lactonase/lactonizing activity of Paraoxonase (PON) (Teiber et al., 2003). It has been postulated that GHB is the primary mediator of GHB/GBL depressant effects. In this sense, GBL is also converted to GHB by PON to mediate the recreational and toxic effects (P Busardo and W Jones, 2015; Krasowski, 2019). PON is a family of enzymes that consists of three members (PON1, PON2, and PON3) with hydrolase activity (Khersonsky and Tawfik, 2005). PONs were recognized as anti-oxidative agents involved in hydrolyzing various toxins such as paraoxon. However, recently it has been proposed that lactones (e.g., GBL) are PONs’ native substrates and PONs are primarily lactonases/lactonizing enzymes, both confirmed by biochemical and structural investigations (Teiber et al., 2003; Harel et al., 2004; Draganov et al., 2005; Hu et al., 2009). Lactones accommodate in the hydrophobic pocket of PONs through hydrophobic interactions, but the size of the lactone ring and ring substituents would determine the binding properties (Khersonsky and Tawfik, 2005; Hu et al., 2009). Since PONs can convert hydroxyl fatty acids to lactones and vice versa, it is suggested that endogenous metabolites could act as native substrates of PONs (Teiber et al., 2003). Similarly, it is also demonstrated that GHB, as an endogenous metabolite, can transform into GBL and vice versa through PON’s activity (Teiber et al., 2003). It is worth noting that peroxisome proliferator-activated receptors γ (PPARγ) regulates the expression of PONs through substances with lactone moiety, which further strengthens the previous observations (Camps et al., 2012; Bedi et al., 2016; Coquant et al., 2020). Exogenous substances with lactone moiety can interfere with the enzymatic activity of PON and its regulation through PPARγ, making them potential treatments for conditions involving PON.

Drug repurposing aims to evaluate the compounds that have already been tested in humans and have demonstrated an acceptable level of safety and tolerability for being utilized outside the scope of their original medical indication (Strittmatter, 2014; Pushpakom et al., 2019). Low risk of failure and reduced time frame and cost make drug repurposing a considerable approach for drug discovery (Pushpakom et al., 2019). Potential candidates for new purposes can be identified through various methods, including investigating shared mechanisms of action of different drugs, comparing their chemical signatures and the relationship to biological activity, and retrospective clinical analysis (Pushpakom et al., 2019). Treatment of addictive substance abuse disorders has already benefited from the drug repurposing approach, and interestingly PPARγ has been among the targets (James et al., 2020; Galaj et al., 2021; Sagheddu et al., 2021). Illuminating the underlying mechanism of GHB/GBL intoxication and addiction is necessary to effectively treat this condition.

Understanding the underlying mechanism of GHB/GBL intoxication and addiction is also of importance in determining the addictive and intoxicating levels of these compounds since they are also produced endogenously. Demonstrating the existence and measuring the level of GHB and GBL in biological fluids and matrices is beneficial in identifying the abusers, detecting possible intoxications, and predicting the clinical course and the response to the treatment. Various methods such as colorimetry (Baumes et al., 2010), chromatography and spectrometry (LeBeau et al., 2000; Schröck et al., 2014; Busardò et al., 2016), capillary electrophoresis (Bortolotti et al., 2004), gravimetric sensing (Brutschy et al., 2013), and fluorescent sensors (Zhai et al., 2013) have been developed and utilized for this purpose. However, due to conversion of GHB and GBL into each other, their relative instability, and involvement of both compounds in addiction and intoxication, determining the level of actual effective compound and interpretation of clinical conditions would become inaccurate, indicating the significance of developing more advanced methods detecting the total true effective compounds.

Herein, we intend to propose a model to complete the mechanism of action of GHB and GBL as recreational drugs and introduce potential candidates with lactone moiety for repurposing to treat GHB and GBL addiction and overdose as a severe and yet unsolved health issue.

### Hypothesis

It has been stated before that GHB mediates the effects of GHB/GBL intoxication; therefore, the role of GBL and its bioactivity are overlooked in the mechanism of GHB/GBL addiction and intoxication. We hypothesize that GBL also plays a critical role in the process of this abusive behavior, addiction, and further intoxications through a specific receptor known as PPARγ. The level of GBL in the body can be increased as a result of GBL consumption or conversion of exogenous GHB to GBL. To be more detailed, GBL, exogenously applied or endogenously produced from the transformation of GHB through enzymatic or spontaneous processes, impose at least part of the addictive or intoxicating effects of GHB or GBL.

This hypothesis completes the current mechanism of action of GHB/GBL intoxication, which suggests that GHB is the primary mediator of GHB/GBL effects through binding to GABA receptors. Through the current hypothesis, we raise the issue that the direct effects of GBL through its specific receptors might be as critical as the effects of GHB in the process of addiction and intoxication, and the latter mechanism of action might not thoroughly explain the clinical effects of GHB and GBL. Introducing PPARγ as a new component in the pathophysiological process of GHB/GBL addiction and intoxication leads to a new target (in addition to PON as a pivotal component) for effective treatments.

In line with our hypothesis, it has been observed that GBL possesses higher potency and faster onset of effects than GHB (Goodwin et al., 2009), indicating that GBL might be the primary mediator of GHB’s primary effects. In this case, either GHB’s effects through GABA receptors might be slower/weaker than GBL’s effects through its receptors, or the GHB conversion into GBL *via* PON takes time. The mediating effect of GBL in abuse of GHB and GBL had been overlooked before since neither a receptor nor a pathway was being specified for it; however, the function of PPARγ as a receptor for substances with lactone moiety [see (Cooley et al., 2010) and (Lin, 2012)] strengthens a new model on the involvement of GBL as the main mediator and a bioactive molecule. Interestingly, in line with this model, PPARγ has been found significant in the process of addiction, and it is currently being targeted for its treatment (Foll et al., 2013; Panlilio et al., 2013; Warden et al., 2016; Matheson and Le Foll, 2020; Quiroga et al., 2021). The new model might pave the road to the discovery of novel antidotes for GHB and GBL intoxication as it restates a new mediator and receptor.

**FIGURE 1:**
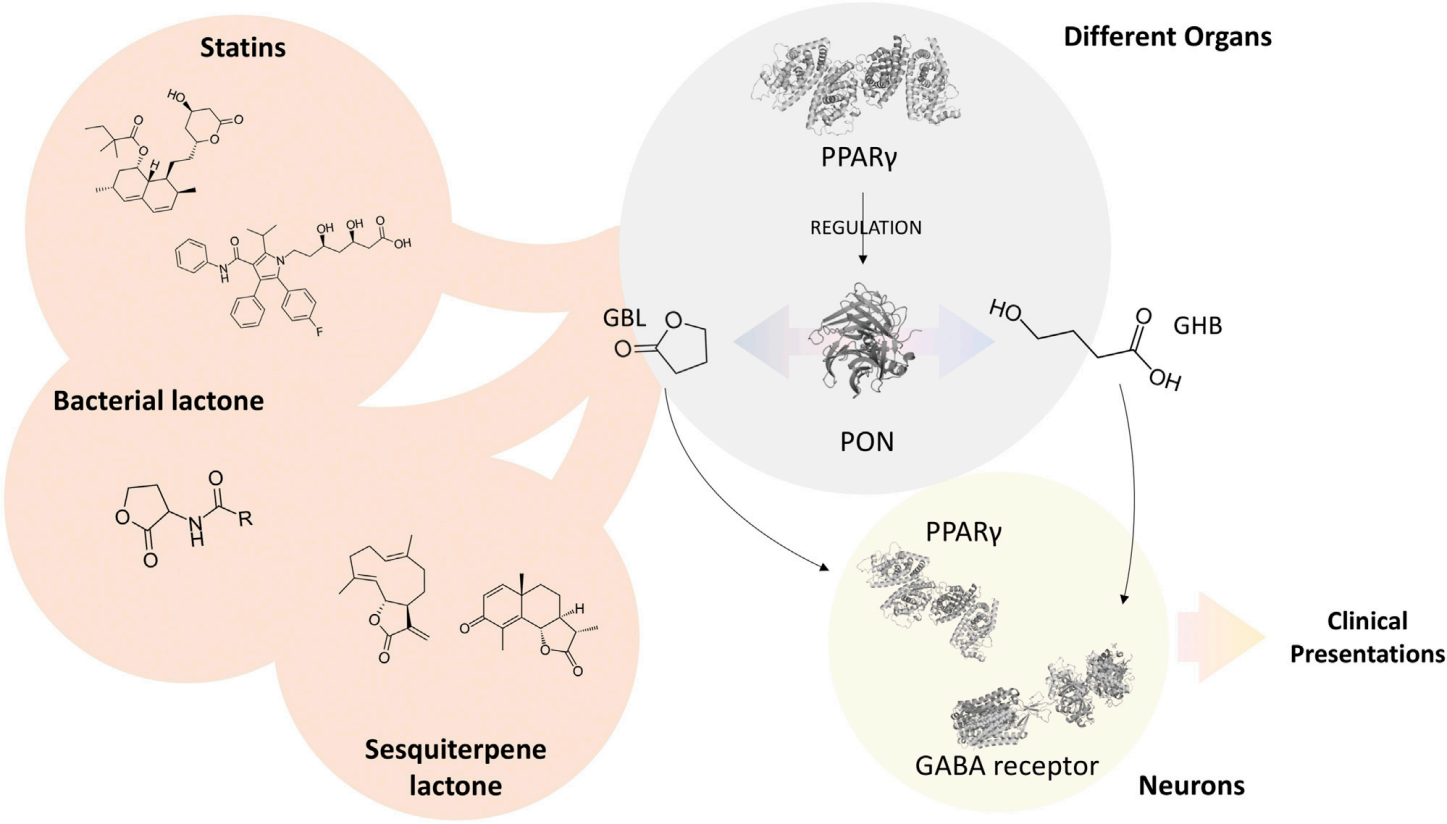
Schematic illustration of hypothesis. The three-dimensional illustrations of proteins were obtained from PDBe hosted by EMBL-EBI (https://www.ebi.ac.uk/pdbe).

Potential antidotes can also be suggested according to the pharmacological mechanisms in which GHB and GBL are involved in. PON, as a lactonase/lactonizing enzyme, is significantly involved in both the activation and deactivation of GHB and GBL. PPARγ, as a regulator of PON, is also playing an essential role in GHB and GBL effects or their elimination. Moreover, PPARγ is involved in metabolic pathways, which might be affected by GHB and GBL. It has been found that lactones are native substrates of PON, and they can specifically bind to PPARγ and change its activity (Draganov et al., 2005; Hu et al., 2009; Cooley et al., 2010; Griffin et al., 2012); Therefore, we hypothesize that substances with lactone moiety can potentially alternate the lactone-acid balance of GHB and GBL, leading to their attenuated activity *via* accommodating the active site of PON and interfering with its function (Figure 1) and affecting PPARγ. In other words, they can act as competitive agonists or antagonists. In addition, they might regulate the expression of PON through the PPARγ-mediated signaling pathway. In the case of excess GBL, *via* inducing the expression of PON through PPARγ or increasing the activity of PON by substances with lactone moiety, it is possible to hinder the destructive effects of GBL through its conversion into GHB, which enters the Krebs cycle (Corkery et al., 2015). Moreover, blocking PPARγ *via* substances with lactone moiety would intercept the PPARγ-meditated effects of GBL. On the other hand, in the case of intoxication with GHB, *via* its partial conversion to GBL through PON, it is possible to reduce the GABA-receptor-mediated effects of GHB. GHB and GBL intoxication not only occur due to abusive behaviors but also in the following of clinical administration of GHB, overuse of cosmetic products, or unsafe contact with industrial materials. Therefore, the current hypothesis might be found effective in treating various patients. Statins, bacterial lactones, and sesquiterpene lactones are some substances with lactone moiety that can be repurposed for the treatment of GHB and GBL addiction and overdose. Interestingly, these substances have been found influential on the expression and activity of PON.

Statins were primarily designed as triglyceride-reducing agents. Statins contain either lactone moiety or its acid formation, which can be converted to lactone *via* PON. Statins are proven to be effective in PON’s expression and activity; however, their overall effect is yet to be determined as the data from different studies are controversial. For instance, simvastatin can modulate PON expression, and it is associated with increased serum PON concentration and activity in a dose-dependent manner (Deakin et al., 2003). In contrast, in another study, it has been demonstrated that pravastatin, simvastatin, or fluvastatin decreased the expression of PON (Gouédard et al., 2003). These differences might arise from different study designs, and further investigations are required to illuminate the actual effects of statins on PON expression and activity. Moreover, the pharmacogenetics profile is a determining factor as the effect of simvastatin is dependent on the pharmacogenetics interactions between the promoter and simvastatin (Deakin et al., 2007). Administration of statins in GHB or GBL intoxication might hinder their effects *via* affecting the level and activity of PON and PPARγ.

Naturally found lactone compounds such as bacterial lactones and sesquiterpene lactones might also find a place for this purpose. Bacterial lactones are the key components of the bacterial quorum sensing process, which have attracted extensive attention due to their novel therapeutic effects in cancer and inflammation. It has been demonstrated that bacterial lactones can be effectively hydrolyzed by the lactonase activity of PONs (Aybey et al., 2015), while they might interfere with its enzymatic function by accommodating the active site. However, their side chains would determine the affinity of PON against these substrates (Aybey et al., 2015). It has also been confirmed that N-3-Oxo-Dodecanoyl-L-Homoserine Lactone, a commonly investigated bacterial lactone, can bind to the PPARγ ligand-binding domain (Cooley et al., 2010). It can act as both agonist and antagonist in the presence of other substances, modulating the transcriptional activity of PPARγ. Sesquiterpene lactones are other natural lactones that originated from plants that all share lactone rings and C15-terpenoids in their chemical structure. Some of the members of this family have already been shown to have similar effects on PON and PPARγ as bacterial lactones (Lin, 2012; Lagoutte et al., 2016; Zhong et al., 2018). The properties of bacterial lactones and sesquiterpene lactones mentioned above make them suitable candidates for being repurposed against GHB or GBL intoxication, acting through modulating the level and activity of PON and PPARγ.

### Evaluation of the hypothesis

In order to evaluate the first hypothesis of the significance of GBL in the process of abusive behavior, investigations on the effects of GBL with simultaneous blockade of GABA receptors and investigations of the effects of GHB with simultaneous blockage of PON and PPARγ is suggested. However, the singular effect of either GHB or GBL is far from expected, and they both probably contribute to the process of abusive behavior and pathophysiology of intoxication and overdose. Moreover, the importance of the PON-PPARγ axis in this process should be evaluated *via* genetic engineering techniques [e.g., knocking out PONs’ genes as in Ref (Rozenberg et al., 2003)] or blockade of PON and PPARγ activity (Lea et al., 2004). The effects of statins, bacterial lactones, and sesquiterpene lactones on the active site of PON and subsequently the kinetic of GHB-GBL conversion in addition to their agonizing/antagonizing effects on PPARγ should be investigated. Colorimetric assays, biosensors, and chromatographic techniques would facilitate the investigation of GHB-GBL conversion *via* PON, as discussed in Ref (Wang et al., 2011). Genetic engineering techniques such as the utilization of fluorescent reporters [as discussed in Ref (Degrelle et al., 2017)] would determine the agonizing/antagonizing effects of proposed substrates. Docking modeling is also of importance for the latter purpose, as in Ref (Hu et al., 2009). *In-vivo* investigations and clinical trials are required as the final steps to translate these substances to the clinic for their newly defined purposes. Measuring the level and activity of PON, resolution of signs and symptoms, the recovery rate of subjects, and the adverse effects are among the essential parameters that are needed to be investigated. It is worth noting that PON and PPARγ are involved in many pathophysiological and physiological pathways, and the aforementioned substances function through various pathways; hence determining the side effects is critical as the proposed hypothesis is not the only axis activated. Moreover, no definite effect of the proposed compounds can be predicted and determined currently, and further enzymological and clinical investigations are required.

### Significance of the hypothesis

GBL is widely accessible due to its industrial applications. It can be found in cosmetic products and alcoholic beverages, which impose health issues if it does not facilitate abusive behaviors. Highlighting the importance of GBL in the process of abusive behavior and further intoxication might lead to two consequences. First, the classification of GBL under the controlled substances category due to its possible direct involvement in the process of addiction and intoxication. Second, the recognition of PPARγ as the potential receptor of GBL and targeting it for therapeutic goals. Moreover, repurposing substances with lactone moiety for the treatment of GHB and GBL intoxication and overdose are of high clinical relevance as the intoxication and overdose are highly probable and occasionally lead to mortality while no antidotes exist. Statins and some sesquiterpene lactones have already been approved for clinical applications; therefore, the repurposing process would take less cost and time, making it an affordable and convenient approach.

## Conclusion

Abuse of GHB and GBL leads to a high mortality rate while no antidote exists. Moreover, the complete mechanism of action of GHB/GBL addiction and intoxication is yet to be discovered, leading to potential novel therapeutic options. While it has been suggested that GHB is the primary mediator of GHB/GBL addiction and intoxication, we hypothesize that GBL might be as critical as GHB in these processes, acting through PPARγ as its receptor, supported by previous studies. In addition, we suggest that repurposing substances with lactone moiety such as bacterial lactones, sesquiterpene lactones, and statins for targeting PPARγ and PON might lead to potential therapeutic options, as these substances would occupy PPARγ and PON active sites specifically.

## Data Availability

The original contributions presented in the study are included in the article/supplementary material, further inquiries can be directed to the corresponding authors.
